# Biological Versus Technical Reliability of Epigenetic Clocks and Implications for Disease Prognosis and Intervention Response

**DOI:** 10.1111/acel.70635

**Published:** 2026-07-29

**Authors:** Raghav Sehgal, Daniel S. Borrus, John Gonzalez, Yaroslav Markov, Albert Higgins‐Chen, Alzheimer's Disease Neuroimaging Initiative

**Affiliations:** ^1^ Department of Psychiatry Yale University School of Medicine New Haven Connecticut USA; ^2^ Department of Pathology Yale University School of Medicine New Haven Connecticut USA

## Abstract

Epigenetic clocks are widely used to estimate biological age and predict health outcomes, but their translational value depends not only on accuracy but also on reliability. Using the TranslAGE platform, we evaluated both technical and biological reliability across 18 DNA methylation–based aging biomarkers, including chronological, mortality, and pace‐of‐aging clocks. Most clocks demonstrated excellent technical reproducibility across replicate assays on EPIC and 450 K arrays. However, several showed sensitivity to experimental factors such as slide position and DNA extraction protocols. In contrast, PC‐based clocks, particularly PCGrimAge and SystemsAge, remained robust across conditions. Biological reliability, assessed across repeated measures collected within short intervals under varying conditions such as meals, stress, and environmental exposures, was substantially lower. Most clocks showed only low to moderate stability which further decreased when adjusting for immune composition. Notably, technical reproducibility did not predict biological reliability. We further show that reliability directly impacts downstream applications. Clocks with higher reliability produced more stable associations with cognitive outcomes and more consistent responses to interventions, while less reliable clocks yielded variable or misleading results. These findings highlight a critical limitation in current epigenetic clocks. Improving biological reliability will be essential for advancing their use in clinical and interventional settings.

## Graphical abstract

Epigenetic clocks accurately measure biological age, but how reliable are they in practice? This study reveals a critical divergence: while clocks are highly consistent across laboratory replicates (technical reliability), their estimates fluctuate significantly in response to everyday events like meals, stress, and pollution (biological reliability). This instability drastically undermines the trustworthiness of clinical predictions and intervention trials, highlighting the urgent need for next‐generation biomarkers resilient to daily physiological noise.

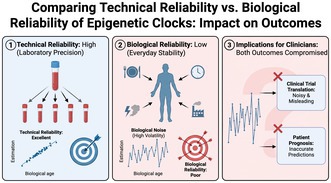

## 1 Introduction

DNA methylation–based (DNAm) aging biomarkers, widely known as epigenetic clocks, have become leading candidates for quantifying biological age (Horvath and Raj 2018; Bell et al. 2019; Teschendorff and Horvath 2025). These measures predict mortality, morbidity, and a range of health outcomes with stronger associations than chronological age alone (Levine et al. 2018; Lu et al. 2022; Belsky et al. 2022). Because they can be derived from a simple blood draw, DNAm biomarkers hold substantial promise for research, clinical trials, and eventually clinical care.

However, despite their rapid adoption, DNA methylation biomarkers face a critical bottleneck: reliability (Higgins‐Chen et al. 2022; Lussier et al. 2024; Tomusiak et al. 2024; Kriukov et al. 2024). For any biomarker to be clinically useful for longitudinal tracking, it must provide consistent and interpretable results across both repeated technical measurements and repeated biological samples (Califf 2018; Mathews et al. 2022). We previously showed that bolstering the technical reliability of epigenetic clocks can increase power and reduce false positives in intervention studies (Higgins‐Chen et al. 2022; Borrus et al. 2024).

Two forms of reliability are essential to distinguish. Technical reliability reflects reproducibility across test–retest measurements from the same blood sample. Variation between technical replicates arises from (1) array platform differences, (2) sample‐level factors such as differences in DNA extraction protocols, storage conditions, slide placement and plate position, low total DNA input quantities, variations in bisulfite conversion efficiency or PCR biases, and array platform differences, or (3) probe‐level differences in probe chemistry, probe hybridization, bead counts, or dye bias (Watkins et al. 2022; Zhang et al. 2024; Nazarenko et al. 2024; Tomo et al. 2025; Bose et al. 2014; Sugden et al. 2020; Dugue et al. 2016; Logue et al. 2017; Pidsley et al. 2016). Biological reliability reflects stability across repeated collections from the same individual across short time frames (hours or days) (Califf 2018; Mathews et al. 2022; Badrick 2021; Mattsson‐Carlgren et al. 2020; Guo et al. 2024; Aliferi 2021). During this interval, events of daily living such as meals, normal levels of stress, circadian variation, or minor environmental exposures may occur. Prior work has demonstrated that many clocks suffer from large technical variability, sometimes producing deviations of nearly a decade between replicates (Higgins‐Chen et al. 2022). Newer clocks such as the PC‐based clocks and DunedinPACE were explicitly designed to improve test–retest reproducibility (Belsky et al. 2022; Higgins‐Chen et al. 2022), but it remains unclear whether such clocks are also more biologically reliable.

This distinction is not trivial. A clock may be technically precise but biologically unstable, yielding values that fluctuate widely in response to acute, reversible, and minor exposures such as a single stressful event or simply different times of day (Apsley et al. 2023; Koncevičius et al. 2024). Such instability risks undermining their clinical and research utility and leads to unexpected sources of confounding, particularly for longitudinal studies. For example, if a meal can dramatically impact a score, then an intervention study that did not control for fasting status pre‐ and post‐intervention may detect changes that are mistakenly attributed to the intervention but actually reflect trivial changes due to meals. Importantly, the impact of reliability on downstream performance, namely whether clocks with higher ICCs (Koo and Li 2016) produce more reproducible prognostic associations and responsiveness estimates, has not been systematically quantified.

Here, we address these gaps by benchmarking the technical and biological reliability of major DNAm clocks spanning five generations: early chronological age predictors (Gen 1), outcome‐trained models (Gen 2), pace‐of‐aging measures (Gen 3), reliable variants of earlier clocks (Gen R), and mechanistically motivated explainable clocks (GenX). These GenX clocks include SystemsAge, OMICmAge, and CausalAge (Sehgal et al. 2025; Ying et al. 2024; Chen et al. 2023). To perform this analysis in a harmonized manner across clocks and datasets, we utilize our TranslAGE platform which we previously used to systematically report the effects of 51 different interventions (Sehgal et al. 2024). Using replicate data, we quantify technical ICCs across arrays, slide positions, and extraction protocols. To probe biological reliability, we test clock stability under short‐term perturbations including meals, stress, pollution exposure, and altitude change. Finally, we directly examine how reliability constrains downstream applications by linking ICCs to the reproducibility of prognostic associations with future cognitive decline and responsiveness to interventions such as a vegan diet. This comprehensive analysis reveals that while most clocks achieve excellent technical reliability, their biological instability limits prognostic and interventional utility, identifying reliability as the central determinant for translating DNAm biomarkers into clinical practice.

## 2 Results

### 2.1 Slide Placement Variations Reduce Technical Reliability for Some Clocks

**FIGURE 1:**
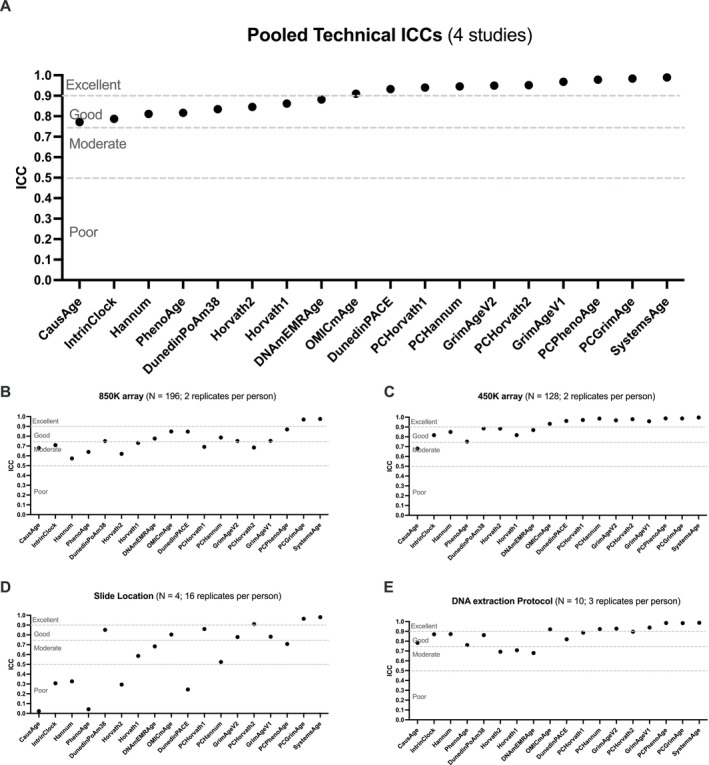
Technical reliability (ICC) of epigenetic clocks across datasets and experimental factors. (A) Pooled technical intraclass correlations (ICCs) for 4 independent studies demonstrate the relative reliability of 18 commonly used epigenetic clocks. Dashed lines denote conventional reliability thresholds (Excellent ≥ 0.90, Good ≥ 0.75, Moderate ≥ 0.50, Poor < 0.50). SystemsAge and principal‐component (PC) versions of established clocks show the highest technical reproducibility. (B–E) Stratified ICCs highlight variation across experimental conditions: (B) 850 K EPIC array (*N* = 196; 2 replicates per person), (C) 450 K array (*N* = 128; 2 replicates per person), (D) slide location effects (*N* = 4; 16 replicates per person), and (E) DNA extraction protocols (*N* = 10; 3 replicates per person). Across all factors, PC‐based measures demonstrate superior reliability, whereas Gen 1 models show greater variability across array type and processing conditions.

We evaluated the technical reproducibility of a broad set of epigenetic clocks across four independent datasets (described in Table 1), encompassing replicate samples processed under different laboratory conditions (Figure 1, Table S1). Overall, the pooled analysis (see Section 4) showed that the majority of clocks achieved excellent technical reliability (ICC > 0.9), with SystemsAge, GrimAge, and PC‐based derivatives consistently ranking among the most stable. Earlier‐generation clocks such as Hannum, PhenoAge, and PCPhenoAge displayed relatively lower but still acceptable levels of reliability, generally in the “good” range (ICC ~0.7–0.8).

**TABLE 1:** Overview of curated DNA methylation datasets included in the analysis.

Study ID	Array type	Type	Individual (replicates)	Age range	Perturbation details
ADNI (Vasanthakumar et al. 2020)	EPICv1	Technical	196 (2)	56–91	—
GSE174422 (Xu et al. 2021)	450 k	Technical	128 (2)	36–75	—
GSE227809—Stress (Apsley et al. 2023)	EPICv1	Biological	34 (4)	18–27	Participants were monitored over a 5‐h period from 11:00 a.m. to 4:15 p.m. Blood samples were collected at four time points: 11:30 a.m., 12:45 p.m., 1:45 p.m., and 4:15 p.m. With the Trier Social Stress Test given at 12 p.m.
GSE227809 (Apsley et al. 2023)	EPICv1	Biological	34 (4)	18–27	Participants were monitored over a 5‐h period from 11:00 a.m. to 4:15 p.m. Blood samples were collected at four time points: 11:30 a.m., 12:45 p.m., 1:45 p.m., and 4:15 p.m. With meal provided at 2 p.m.
GSE105123 (D'Alessandro et al. 2016)	450 k	Biological	21 (4)	19‐23	Participants were studied at sea level (130 m) and during high‐altitude exposure at 5260 m in Bolivia. Samples were taken at Sea level, after climbing 5250 m, 2 samples on Day 1 (30 min apart) and 1 sample on Day 7.
GSE56553 (Jiang et al. 2014)	450 k	Biological	16 (3)	19–46	Blood samples were collected prior to exposure and subsequently at 6 and 30 h post‐exposure.
GSE250556 (Butler et al. 2025)	EPICv1	Technical	4 (16)	24‐66	Each subject had eight independent blood‐derived DNA extractions, each split into technical replicates that underwent separate bisulfite conversion, amplification, and array processing, alongside a matched pooled‐DNA replicate design.
Zenodo1285774 (Hjorthaug et al. 2018)	450 k	Technical	10 (3)	15–66	Three DNA extraction methods were compared, including two automated approaches (magnetic bead–based and isopropanol precipitation) and a manual organic extraction protocol.

*Note:* This table summarizes the publicly available datasets selected after manual review and curation of primary study documentation and supplementary materials. For each dataset, we report the study identifier, array platform, replicate type (technical or biological), and the number of unique individuals with the corresponding number of replicates per individual (shown in parentheses). These datasets were curated to enable systematic evaluation of variability and reproducibility across platforms and replicate structures.

When stratified by array platform, both the Illumina 850 K (*N* = 196; 2 replicates per person) and 450 K (*N* = 128; 2 replicates per person) arrays demonstrated high reproducibility across most clocks, reinforcing that array type alone does not substantially compromise technical stability. However, further sensitivity analyses revealed notable drops in reliability under specific laboratory conditions. In particular, clock estimates exhibited marked variability when replicates differed by slide location (*N* = 4; 16 replicates per person), where nine clocks fell below the threshold of “good” reliability and six clocks dipped in “moderate” levels or worse. Similarly, while the majority of clocks remained stable under different DNA extraction protocols (*N* = 10; 3 replicates per person), a subset of three clocks displayed diminished reproducibility, highlighting that upstream sample processing can introduce additional noise.

Taken together, these results demonstrate that while epigenetic clocks are generally technically robust across arrays and replicates, their performance can degrade under more subtle laboratory perturbations such as slide placement. These findings emphasize the need for careful attention to laboratory workflows when applying clocks in clinical or research settings, while also suggesting that biological rather than technical variation likely accounts for much of the observed instability in downstream prognostic and interventional applications.

### 2.2 Short‐Term Exposures Reduce Biological Reliability of Epigenetic Clocks

**FIGURE 2:**
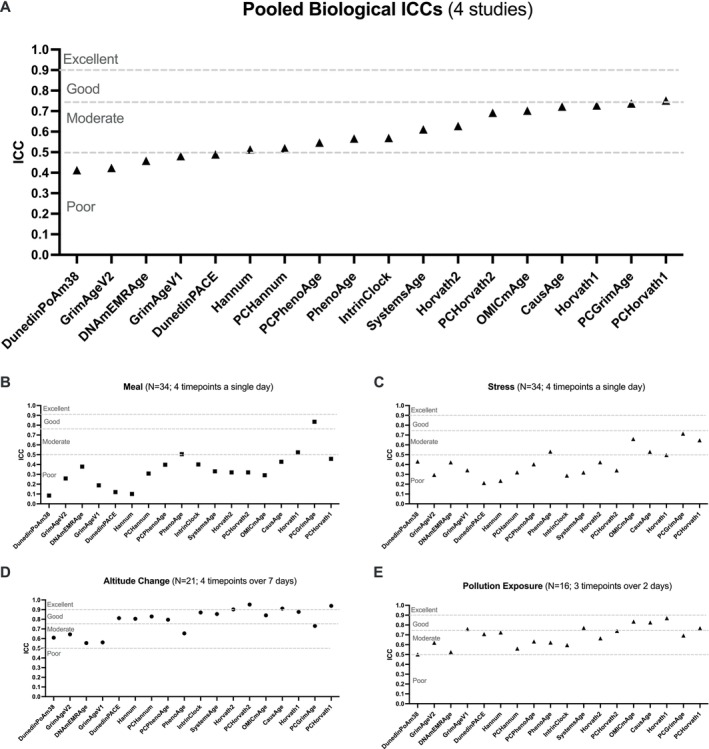
Biological reliability (ICC) of epigenetic clocks across short‐term physiological perturbations. (A) Pooled biological intraclass correlations (ICCs) from four independent studies measuring repeated biological samples indicate variable short‐term reproducibility among 18 epigenetic clocks. Dashed lines denote standard ICC reliability thresholds (Excellent ≥ 0.90, Good ≥ 0.75, Moderate ≥ 0.50, Poor < 0.50). Multi‐system and principal‐component (PC) clocks such as SystemsAge, PCPhenoAge, and PCGrimAge show greater biological stability than single‐tissue or unadjusted versions. (B–E) Stratified ICCs by acute or environmental challenge illustrate differential within‐person biological variability: (B) postprandial effects after a standardized meal (*N* = 34; 4 timepoints in 1 day), (C) acute psychosocial stress (*N* = 34; 4 timepoints in 1 day), (D) altitude acclimatization (*N* = 21; 4 timepoints over 7 days), and (E) short‐term air pollution exposure (*N* = 16; 3 timepoints over 2 days). Biological stability was highest under chronic or environmental exposures (altitude, pollution) and lowest under acute perturbations (meal, stress), underscoring the importance of temporal and physiological context in interpreting DNA methylation–based aging biomarkers.

In contrast to the consistently high technical reproducibility of epigenetic clocks, their *biological reliability* across repeated measures within the same individuals was substantially lower after single short‐term events such as meals, stress, pollution, and altitude change (Figure 2, Table S2). We first tested for systematic changes up or down for the clocks, and found that only altitude change after 7 days caused a systematic shift. We removed time points after this period for altitude change, so that remaining analyses would reveal only random biological noise shifts. Pooled estimates (see Section 4) from four independent studies revealed that most clocks achieved only “moderate” to “good” reliability (ICC ≈ 0.4–0.7), with no clock reaching the “excellent” range. Clocks such as GrimAge (versions I and II), DNAmEMRAge, and DunedinPACE generally clustered near the lower end of this spectrum, while PC‐based and later‐generation clocks (e.g., PCGrimAge, SystemsAge, OMICmAge) performed somewhat better but still fell short of the reproducibility observed in technical replicates.

Condition‐specific analyses highlighted the sensitivity of clocks to short‐term biological fluctuations. Following a single meal (*N* = 34; 4 timepoints within a day), reliability dropped markedly, with many clocks performing only in the “moderate” or “poor” range, suggesting strong acute biological variability. Stress exposure (*N* = 34; 4 timepoints within a day) produced a similar effect, with several widely used clocks including DunedinPACE and GrimAge falling into the “poor” reliability range. Pollution exposure (*N* = 16; 3 timepoints over 2 days) also induced sharp drops in stability, reinforcing the susceptibility of clock estimates to short‐term environmental perturbations. By contrast, changes across altitude (*N* = 21; 4 timepoints over 7 days) showed comparatively higher stability, with several clocks approaching “good” reliability, suggesting that slower or more systemic physiological adaptations may exert less volatility on methylation‐based age measures than acute perturbations.

Together, these findings demonstrate that while epigenetic clocks are technically robust, their biological instability under everyday exposures: including diet, stress, and environmental conditions poses a significant limitation.

### 2.3 Immune Cell Adjustment Decreases Biological Reliability in Epigenetic Clocks

Because blood cell composition is a major source of variation in blood DNA methylation data, we hypothesized that adjusting for immune cell fractions would remove biological noise and improve the biological reliability of epigenetic clocks. Specifically, we expected that regressing out estimated immune cell proportions would increase biological intraclass correlation coefficients (ICCs) by reducing variability attributable to fluctuations in circulating cell populations.

**FIGURE 3:**
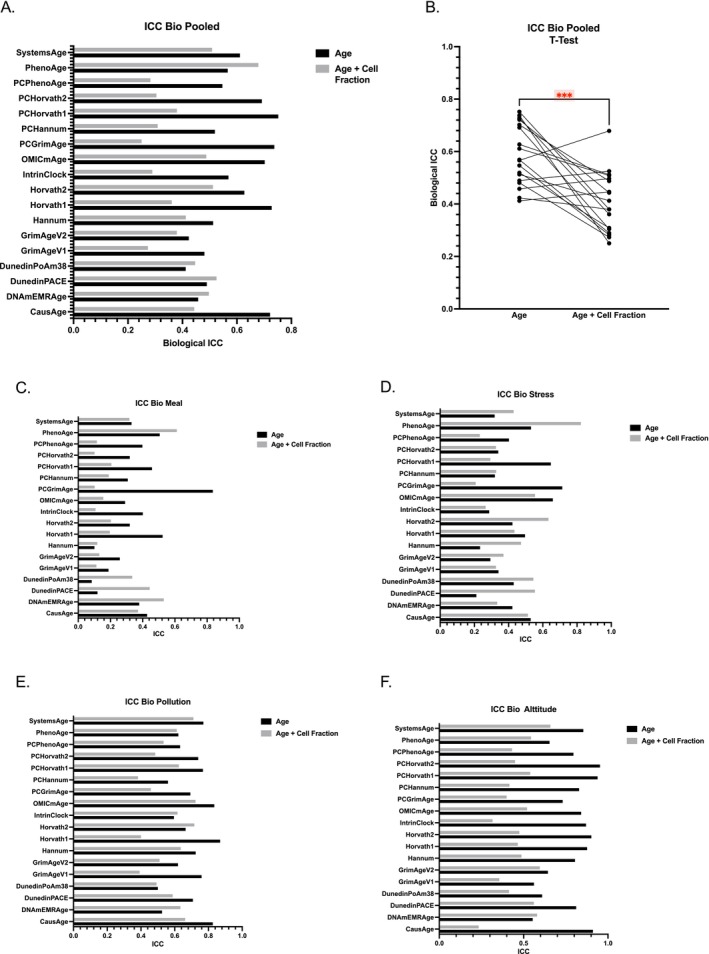
Adjustment for immune cell composition decreases the biological reliability of epigenetic clocks. Biological intraclass correlation coefficients (ICCs) were calculated for epigenetic clocks before (Age) and after adjustment for nine estimated immune cell fractions (Cell Fraction + Age). (A) Across all datasets combined, adjustment for immune cell composition resulted in lower biological ICCs for nearly every clock examined. (B) Paired comparison of biological ICCs across clocks demonstrates a significant reduction following immune cell adjustment (****p < 0.0001). (C–F) The same pattern was observed across individual intervention paradigms, including meal consumption (C), acute stress (D), physical exhaustion (E), and high‐altitude exposure (F). Contrary to the expectation that removing immune cell variation would stabilize epigenetic age measurements and increase biological reliability, adjustment for cell fractions consistently reduced ICCs. These findings suggest that immune cell composition contributes meaningful biological signal to epigenetic clock measurements and may play an important role in maintaining the reproducibility of biological aging estimates across physiological perturbations.

Contrary to this expectation, adjustment for immune cell fractions consistently reduced biological reliability across nearly all clocks examined (Figure 3, Table S3). In the overall dataset (Figure 3A), biological ICCs were substantially lower after adjustment for immune cell composition compared with models adjusted only for chronological age. This pattern was observed across first‐generation chronological age clocks, second‐generation mortality and healthspan predictors, and next‐generation biological aging measures. The only dramatic increase in ICC was seen for PhenoAge with slight increases in DunedinPACE and DNAmEMRAge, although all of these were within margin of error. In general, though, the reduction in biological ICC was highly consistent across clocks, resulting in a significant overall decrease in reliability following immune cell adjustment (Figure 3B; *p* < 0.001).

To further investigate this effect, we evaluated biological reliability across four intervention paradigms: meal consumption, acute stress, pollution exhaustion, and high‐altitude exposure (Figure 3C–F). Across all paradigms, immune cell adjustment generally reduced biological ICCs relative to age‐adjusted models alone. The largest decreases were observed in the meal and altitude change conditions, where several clocks exhibited marked reductions in biological reliability after cell‐fraction correction. It is worth noting that reduced reliability did not stand true for all clocks and we observed improvements in ICC in clocks such as DunedinPACE and Phenoage. Similar declines in reliability were observed following pollution and altitude exposure, though to a lesser degree.

These findings suggest that immune cell variation contributes substantially to the biological signal captured by epigenetic clocks. Rather than acting solely as a source of unwanted variability, changes in immune cell composition appear to reflect meaningful biological processes that are reproducibly detected by DNA methylation aging biomarkers. Consequently, regressing out immune cell fractions may remove biologically relevant information and reduce the ability of epigenetic clocks to consistently capture physiological responses. Overall, our results indicate that immune‐cell dynamics represent an important component of epigenetic age measurements and that routine adjustment for cell composition may inadvertently diminish biological reliability.

### 2.4 Technical Reliability Is Not Correlated With Biological Reliability of Epigenetic Clocks

**FIGURE 4:**
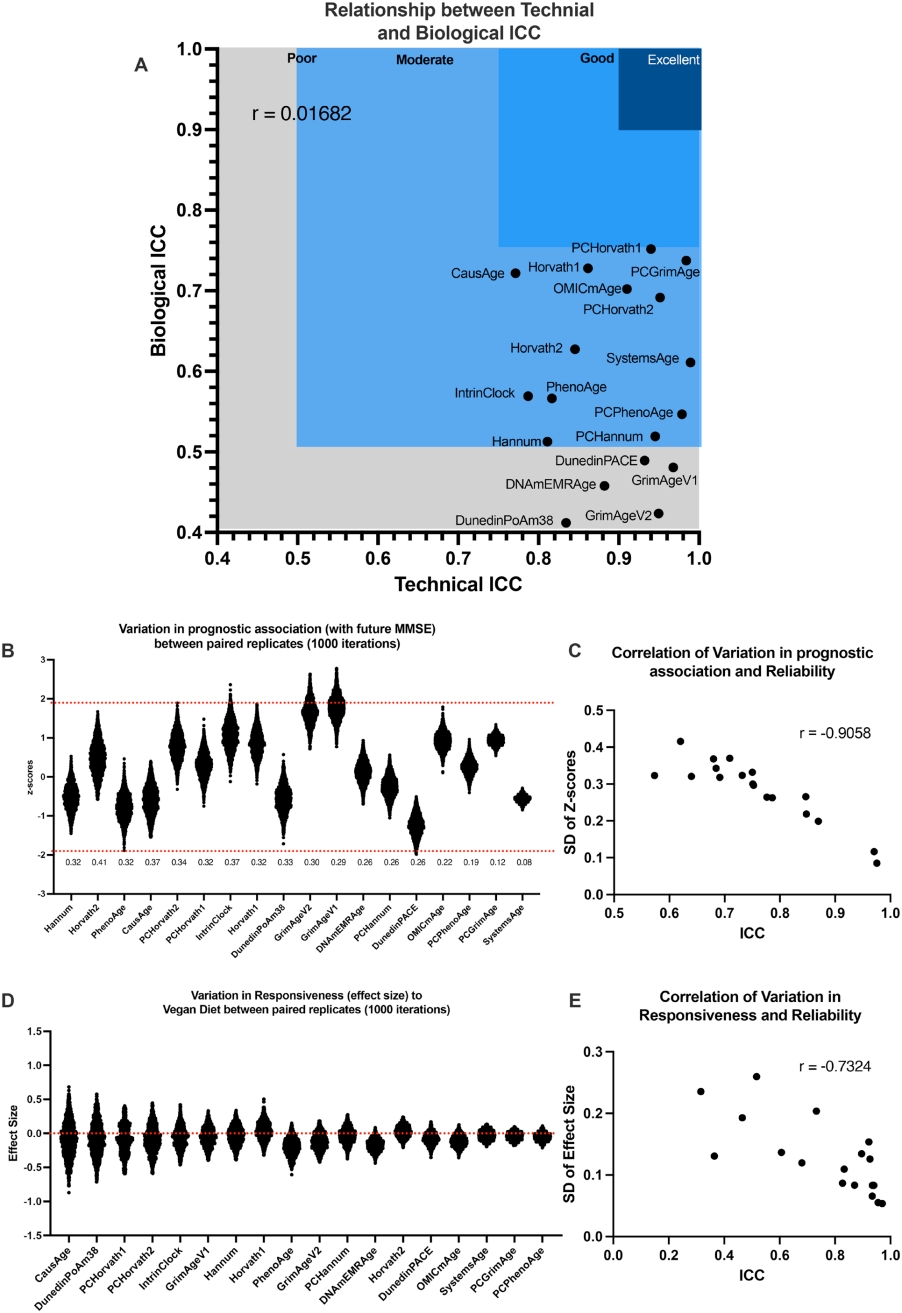
Relationship between technical reliability, biological stability, and downstream analytic variability of epigenetic clocks. (A) Relationship between pooled technical and biological intraclass correlations (ICCs) across 18 epigenetic clocks demonstrates that technical and biological reproducibility are largely independent (*r* = 0.0168). Principal‐component (PC) clocks (e.g., PCPhenoAge, PCGrimAge, PCHorvath1, Systems Age) show high reproducibility in both dimensions, while first‐generation clocks and Dunedin‐based measures exhibit lower stability. (B) Variation in prognostic associations with future Mini‐Mental State Examination (MMSE) scores across 1000 resampling iterations illustrates greater variability for lower‐ICC clocks. (C) The standard deviation of prognostic *Z*‐scores is strongly inversely correlated with reliability (*r* = −0.9058), indicating that clocks with higher ICCs yield more consistent effect estimates across replicates. (D) Variation in responsiveness (effect size) to a vegan diet intervention across 1000 paired resampling iterations reveals that unreliable clocks show greater dispersion in estimated effects. (E) Standard deviation of responsiveness effect sizes is also inversely correlated with ICC (*r* = −0.7324). Together, these analyses show that clocks with higher technical and biological reliability produce more stable and reproducible estimates of both prognostic and interventional outcomes.

We examined the relationship between technical and biological reliability across clocks (Figure 4A). While nearly all clocks achieved high technical ICCs, their corresponding biological ICCs were substantially lower, with no clear correlation between the two metrics (*r* = 0.0168). For example, SystemsAge and PC‐based clocks (e.g., PCGrimAge, PCHorvath1 & 2) were among the most technically stable and showed comparatively higher biological reliability, yet widely used measures such as GrimAgeV2 and DunedinPACE remained technically excellent but biologically unreliable.

### 2.5 Clock Instability Undermines Prognostic Power and Intervention Responsiveness

Next, we evaluated how clock reliability impacts their utility in prognostic and interventional settings (Figure 4B–D, Table S4). Using repeated resampling of paired technical replicates (1000 iterations, see Section 4), we quantified the stability of associations between clocks and two key outcomes: (1) prognostic prediction of future cognition, measured by Mini‐Mental State Examination (MMSE) (Tombaugh and McIntyre 1992), and (2) responsiveness to intervention, estimated as effect sizes for a vegan diet exposure.

Clocks with lower reliability displayed strikingly wide variability in their associations with future MMSE scores. Across resampling iterations, low‐ICC clocks such as Hannum, Horvath, PhenoAge, and CausAge produced highly unstable effect estimates, with *z*‐scores fluctuating across the full significance spectrum, rendering any observed association untrustworthy. In contrast, high‐reliability clocks including SystemsAge, PCGrimAge, PCPhenoAge, and DunedinPACE produced far narrower distributions centered around consistent *z*‐scores. Quantitatively, reliability strongly predicted the stability of prognostic associations: the standard deviation of *z*‐scores across replicates correlated negatively with Biological ICC (*r* = −0.91). This demonstrates that if you observe prognostic power in a low‐reliability clock, that signal cannot be trusted. Conversely, high‐reliability clocks yield reproducible prognostic signals that are dependable for clinical interpretation.

We applied our resampling technique to an intervention dataset with technical replicates to measure the impact of technical reliability on biomarker responsiveness, and found a similar trend. When estimating the impact of vegan diet exposure, less reliable clocks (Hanum, Horvath, Phenoage, CausAge and more) showed large variability in effect sizes across replicates, often flipping direction or yielding inflated effect sizes. In contrast, biologically stable clocks (PCGrimAge, PCPhenoAge, SystemsAge, DunedinPACE) maintained consistent effect size estimates even under repeated resampling. The correlation between Biological ICC and variability in intervention responsiveness (*r* = −0.73) confirmed that clock instability undermines the trustworthiness of intervention effects. High‐reliability clocks offer stable, reproducible intervention signals suitable for rigorous clinical trials.

Together, these analyses establish reliability as a critical prerequisite for translational application of epigenetic clocks. Clock instability is the enemy of trustworthy results: unstable clocks can generate spurious or inconsistent associations and misleading intervention estimates, leading to false conclusions in clinical or research contexts. Conversely, clocks with higher biological reliability provide stable, reproducible signals that clinicians and researchers can confidently rely upon in longitudinal studies and interventional trials.

## 3 Discussion

The promise of epigenetic clocks lies in their ability to act as prognostic and interventional biomarkers, yet our results show that their utility is critically constrained by reliability (Higgins‐Chen et al. 2022; Cummings and Kritchevsky 2022). While discussions of biomarkers often emphasize accuracy and predictive value, the consistency of measurement across both technical and biological contexts is equally essential for clinical translation and longitudinal studies (Cummings and Kritchevsky 2022). This study provides the most comprehensive evaluation to date of both technical and biological reliability across multiple generations of DNA methylation (DNAm) clocks, and directly demonstrates how reliability governs prognostic and interventional performance.

Our findings confirm that technical reliability is largely a solved problem. Across four independent datasets, most clocks demonstrated excellent reproducibility in replicate assays, with PC‐based clocks such as SystemsAge consistently ranking among the most stable. Technical robustness also translated into greater consistency in prognostic predictions, as shown by narrower distributions of associations with cognitive decline and brain volume loss. These results underscore that technical noise, while once a significant limitation for early clocks such as Horvath1, Hannum, and PhenoAge, has been effectively addressed in Generation Reliable models.

By contrast, biological reliability remains a major obstacle. We found that repeated measures taken under common short‐term perturbations, including meals, stress, and pollution exposure, caused substantial fluctuations in epigenetic age estimates, with most clocks falling into only moderate reliability at best (Higgins‐Chen et al. 2022; Komaki et al. 2022; Galkin et al. 2023). Importantly, these are the types of perturbations that would occur during the course of everyday life, making them common confounding events. Adjusting for immune cell composition, we saw that biological reliability dropped significantly. Critically, technical reproducibility did not predict biological stability: some of the most technically robust clocks, such as GrimAgeV2 and DunedinPACE, were among the most biologically fragile. This dissociation reveals that laboratory precision alone is not sufficient to ensure a biomarker's clinical utility.

The consequences of biological instability are not merely theoretical. Our analyses show that clocks with lower reliability produce unstable prognostic predictions and inconsistent intervention responsiveness. Associations with cognitive decline and dietary exposures varied widely across replicate analyses for unreliable clocks, raising the risk of spurious or misleading findings. We previously warned about false positive intervention results in clocks with low reliability, especially chronological age clocks (Borrus et al. 2024; Sehgal et al. 2024). Conversely, clocks with higher reliability, particularly PCGrimAge and SystemsAge, provided stable signals across both prognostic and interventional contexts. These results highlight reliability as the central determinant of translational readiness. Importantly, there was no relationship between technical reproducibility and biological stability of clocks, reinforcing the importance of distinguishing between laboratory precision and biological robustness when evaluating clock performance.

It is worth mentioning that these results should be taken in context of other available biomarkers. Even though we see moderate biological reliability from the best Generation Reliable DNAm biomarkers, they are still better or comparable to several well‐known biomarkers currently in clinical practice or used as surrogate endpoints. For example, Aβ40 shows a time‐of‐day varying ICC of 0.76, Aβ42 an ICC of 0.88, and GFAP an ICC of 0.83 (Della Monica et al. 2024). Clinical biomarkers like hematocrit have been reported with ICCs as low as 0.61 pre‐ and post‐hydration (Doherty et al. 2024). Moreover, meal consumption or fasting can alter protein biomarkers such as insulin (ICC = 0.75), C‐peptide (ICC = 0.66), and free IGF‐1 (ICC = 0.55) (Murphy et al. 2016). Other widely used proteomic biomarkers show even lower stability in response to perturbations such as exercise training, including BDNF (ICC = 0.26), IL‐1β (ICC = 0.65), IL‐6 (ICC = 0.0), and IFN‐γ (ICC = 0.11) (Reichel et al. 2023). These comparisons suggest that while DNAm biomarkers are biologically unstable, their reliability is not unusually poor when judged against other biomarker types, and in some cases DNAm biomarkers are superior.

This study has several limitations. First, while we examined reliability across multiple datasets and perturbations, our analyses remain constrained to specific populations and conditions. Larger, more diverse cohorts are needed to test whether the observed patterns generalize across ancestries, age groups, and disease states. Second, some may argue that the biomarker changes we observe after events of daily life such as meals, stress, and altitude change may be bona fide changes in the aging process. However, we do not think it is plausible that such minor single events should truly age or de‐age a person by multiple years. Even if these events did have such a large effect, it still highlights the importance of identifying methods to separate their effects from intervention treatment effects occurring in the same period. Third, the biological perturbations we tested were relatively short‐term and acute. Longitudinal studies spanning years are required to determine whether biological instability resolves over longer intervals or reflects intrinsic noise in methylation dynamics. Fourth, while we identified correlations between reliability and prognostic or interventional stability, causal mechanisms underlying biological instability remain unclear. Immune cell dynamics, stress‐related pathways, and metabolic fluctuations are likely contributors, but further mechanistic studies will be necessary (Tong et al. 2024). Fifth, the biological events we examined did not have control groups of similar individuals examined at the exact same time, so it remains unclear whether the instability is due to the biological event or simply due to taking blood samples at different times. Sixth, we used EPICV1 and 450 K datasets for technical reliability which are no longer used, future studies should perform this similar analysis in EPICV2 datasets. Seventh, the technical reliability estimates are based solely on within‐study, within‐laboratory replicates and do not capture additional sources of variability such as cross‐laboratory differences or reagent lot variation, which are known contributors to analytical variability in DNA methylation assays. Eighth, three of the four biological reliability datasets are derived from relatively young participants (aged 18–27), which limits the generalizability of the observed biological reliability estimates to older populations where DNA methylation clocks are more commonly applied in clinical and translational settings. Future studies should explicitly evaluate whether these reliability patterns hold across older age groups and more clinically relevant populations. Finally, although we focused on widely used clocks, the field is rapidly evolving, and newer models may address some of the limitations highlighted here.

In conclusion, our findings demonstrate that while epigenetic clocks now achieve excellent technical reproducibility, biological instability remains a central limitation to their clinical translation. Reliability is the key constraint shaping prognostic and interventional utility, and efforts to develop next‐generation biomarkers must prioritize not only technical robustness but also resilience to biological variability.

## 4 Methods

### 4.1 Analysis Pipeline—TranslAGE Reliability

#### 4.1.1 Step 1: Data Curation

We began by compiling a comprehensive list of publicly available reliability datasets. Publicly available datasets were sourced from Gene Expression Omnibus (GEO) and the European Molecular Biology Laboratory (EMBL) repositories and other public data repositories. Datasets were selected on the basis of the following criterion: (1) DNAm data is either for technical or biological replicates, (2) tissue of origin for the DNAm data is blood (ideally PBMCs), (3) study passes quality checks. For each study, we curated extensive study‐level metadata, including the following key variables:

- Type of technical or biological variability: Identifying the different possible variations that could be possible given a set of replicate samples.
- Demographic data: Capturing demographic distribution to test reliability across a wide gamut of age, sex, and other demographic parameters.

These metadata were manually extracted from study documentation and supplementary materials to ensure accuracy. After careful curation, the following datasets were curated for analysis as described in Table 1.

Data used in the preparation of this article were obtained from the Alzheimer's Disease Neuroimaging Initiative (ADNI) database (adni.loni.usc.edu). The ADNI was launched in 2003 as a public‐private partnership, led by Principal Investigator Michael W. Weiner, MD. The original goal of ADNI was to test whether serial magnetic resonance imaging (MRI), positron emission tomography (PET), other biological markers, and clinical and neuropsychological assessment can be combined to measure the progression of mild cognitive impairment (MCI) and early Alzheimer's disease (AD). The current goals include validating biomarkers for clinical trials, improving the generalizability of ADNI data by increasing diversity in the participant cohort, and providing data concerning the diagnosis and progression of Alzheimer's disease to the scientific community. For up‐to‐date information, see adni.loni.usc.edu.

#### 4.1.2 Step 2: Metadata Standardization

We utilized a standardized cleaning script that was adapted for each dataset. We standardized the variables across all studies to create uniform, comparable datasets. This harmonization process involved:

- Standardized columns: We ensured that all datasets contained consistent variables, including Age, Sex, Sample ID, Individual ID, and Replicate condition. This step allowed for consistent cross‐study analysis.
- Sample identification: To maintain data integrity, each sample was assigned a unique Sample ID and linked to an Individual ID, ensuring that repeated measurements from the same individual could be tracked longitudinally.

This harmonization facilitated subsequent analyses, allowing for robust comparisons between different studies and intervention types.

#### 4.1.3 Step 3: Clock Calculation

For each of the curated datasets, we calculated over 18 DNA methylation (DNAm) biomarkers using the MethylCIPHER 2.0 package. This package allows for the computation of various methylation‐based aging and health biomarkers. These biomarkers were calculated for each sample based on the methylation data available in each study.

#### 4.1.4 Step 4: Age‐Residualized Scores

We processed the biomarkers by age‐residualizing them in each dataset. Age‐residualization was performed to control for the natural variation in DNAm biomarkers due to chronological age. The procedure involved linear regression. We used age as the independent variable and each DNAm biomarker as the dependent variable to calculate the residuals. These age‐residuals represent the portion of the biomarker that is not explained by chronological age.

#### 4.1.5 Step 5: ICC Calculation

We performed Intraclass Correlation Coefficient (ICC) analysis to evaluate the reliability of each epigenetic clock. ICC measures the consistency or reproducibility of measurements, with higher values indicating greater reliability. The analysis was conducted using the irr (0.84.1) package in R, specifically using the icc() function to compute the ICC for each clock across the paired samples. Function irr::icc(data, model = “twoway”, type = “agreement”, unit = “single”). We used a *p*‐value cut off of 0.05 to determine non‐significant ICCs (Shrout and Fleiss 1979).

### 4.2 ICC Pooling Methodology

ICCs were pooled across multiple datasets using a comprehensive meta‐analytical approach with both fixed‐effects and random‐effects models. The pooling procedure involved the following steps:

- Fisher's *Z*‐transformation: Individual ICC values were transformed using Fisher's *z*‐transformation [*z* = 0.5 × ln((1 + ICC)/(1 − ICC))] to normalize their distribution and stabilize variance, with values clipped to [−0.999, 0.999] to ensure valid transformations.
- Weighting scheme: Study‐specific weights were calculated as the inverse of the variance (1/SE^2^), where standard error was estimated using SE = 1/√(*n* − 3) based on the effective sample size (minimum of subjects and observations).
- Random‐effects model: The DerSimonian‐Laird method was employed to account for between‐study heterogeneity, incorporating tau‐squared (*τ* ^2^) estimates of between‐study variance into the weighting scheme.

Separate meta‐analyses were conducted for biological and technical replicability datasets, with 95% confidence intervals calculated for both fixed and random‐effects estimates. Results showed substantial heterogeneity (*I* ^2^ > 50%) for most epigenetic clocks, justifying the use of random‐effects models as the primary pooling approach.

### 4.3 Cell Composition and Its Effects on Biological Reliability Analysis

To evaluate the contribution of immune cell composition to the biological reliability of epigenetic clocks, blood immune cell fractions were estimated from DNA methylation data using the EpiDISH (Epigenetic Dissection of Intra‐Sample Heterogeneity) R package. The centDHSbloodDMC.m reference panel and robust partial correlation (RPC) algorithm were used to estimate the proportions of seven immune cell populations: CD4^+^ T cells, CD8^+^ T cells, natural killer (NK) cells, B cells, monocytes, neutrophils, and eosinophils.

The adjustment involved linear regression as described in Step 4 previously although this time with each DNAm biomarker as the dependent variable and chronological age, CD4^+^ T cells, CD8^+^ T cells, natural killer (NK) cells, B cells, monocytes, neutrophils, and eosinophils as independent variables. Residuals from each model were extracted and used as the corresponding adjusted clock values. Biological reliability was quantified using intraclass correlation coefficients (ICCs) calculated from repeated measurements obtained under biological perturbations. ICCs were computed for the original clock values, age‐adjusted clock values, and age‐ and immune cell‐adjusted clock values. Comparisons were performed across all biological reliability datasets combined using the pooling of ICC methodology above.

### 4.4 Multiverse Analysis to Assess Effects of Technical Noise on Prognostic Associations

To evaluate the impact of technical noise on morbidity associations, we performed a multiverse analysis by simulating various scenarios where one technical replicate from each pair was randomly selected for the association analysis. We performed 1000 such association analyses for each epigenetic clock by randomly selecting one of the two technical replicates for each individual in the ADNI dataset (Petersen et al. 2010). For the ADNI prognostic analysis, we used the ADNI 1/GO/2 merged data release and adjusted for age and sex in the model. The goal was to evaluate how technical noise might affect the variability in prognostic association *z*‐scores across multiple analyses.

1. Random selection of replicates: For each of the 1000 simulated association analyses, we randomly selected one of the two technical replicates for each individual. This process was repeated independently for each simulation to introduce variability in the input data.
2. Association analysis for each simulation: We then performed the association analysis using the selected replicates in each simulation, calculating the *z*‐scores for disease associations with each epigenetic clock.
3. Comparing *z*‐score distributions: After completing all 1000 simulations, we compared the distribution of the resulting *z*‐scores for each epigenetic clock. This allowed us to quantify the potential variation in association results due to technical noise.

This approach allowed us to systematically explore how technical variation between replicates could influence disease association outcomes for each clock, providing insight into the robustness of the associations in the presence of technical noise.

### 4.5 Multiverse Analysis to Assess Effects of Technical Noise on Responsive Associations

To extend the assessment of variability in intervention responsiveness due to technical replicate selection, we conducted a sensitivity analysis across the vegan diet intervention dataset (NCT05297825, 8 weeks of intervention, PMID: 39069614); this subset analysis comprised 8 individuals with 2 technical replicates each.

For each DNAm biomarker, the analysis proceeded as follows.

#### 4.5.1 Responsiveness Estimation Across Technical Replicates

We re‐estimated intervention effect sizes using one of the two technical replicates per sample, and then repeated the analysis using the alternate replicate, yielding two sets of effect size *z*‐scores for each biomarker.

#### 4.5.2 Calculation of Replicate‐Dependent Differences in Responsiveness

We computed the difference between effect size *z*‐scores derived from the primary replicate and those derived from the alternate replicate (Δ*z* = *z*_primary − *z*_alternate), providing a measure of sensitivity of inferred responsiveness to replicate selection.

#### 4.5.3 Cross‐Dataset Comparison of Responsiveness Variability

We compared the distribution of Δ*z* values across DNAm biomarkers within and across all intervention datasets, including the vegan diet cohort (8 samples, 2 replicates each) to evaluate whether replicate‐driven variability in responsiveness was consistent across distinct biological perturbation contexts.

This framework allowed us to quantify how technical replicate selection influences estimated intervention responsiveness across multiple intervention paradigms.

## Author Contributions

R.S. and A.H.‐C. conceived the project. R.S. conceived the study design. R.S. and D.B. performed reliability analysis. R.S., D.B., J.G., Y.M., and A.H.‐C. built the pipeline for TranslAGE. R.S. performed study data and metadata curation for reliability datasets. Y.M. processed raw ADNI DNA methylation data. R.S. wrote the manuscript, and all authors reviewed and contributed to the manuscript.

## Funding

This work was supported by the National Institute on Aging (NIA:1R01AG065403), the Impetus Grant, the Gruber Science Fellowship at Yale University, and the Thomas P. Detre Fellowship Award in Translational Neuroscience Research from Yale University.

## Disclosure

Data collection and sharing for the Alzheimer's Disease Neuroimaging Initiative (ADNI) is funded by the National Institute on Aging (National Institutes of Health Grant U19AG024904). The grantee organization is the Northern California Institute for Research and Education. In the past, ADNI has also received funding from the National Institute of Biomedical Imaging and Bioengineering, the Canadian Institutes of Health Research, and private sector contributions through the Foundation for the National Institutes of Health (FNIH) including generous contributions from the following: AbbVie, Alzheimer's Association, Alzheimer's Drug Discovery Foundation, Araclon Biotech, BioClinica Inc., Biogen, Bristol‐Myers Squibb Company, CereSpir Inc., Cogstate, Eisai Inc., Elan Pharmaceuticals Inc., Eli Lilly and Company, EuroImmun, F. Hoffmann‐La Roche Ltd. and its affiliated company Genentech Inc., Fujirebio, GE Healthcare, IXICO Ltd., Janssen Alzheimer Immunotherapy Research & Development LLC, Johnson & Johnson Pharmaceutical Research & Development LLC, Lumosity, Lundbeck, Merck & Co. Inc., Meso Scale Diagnostics LLC, NeuroRx Research, Neurotrack Technologies, Novartis Pharmaceuticals Corporation, Pfizer Inc., Piramal Imaging, Servier, Takeda Pharmaceutical Company, and Transition Therapeutics.

## Conflicts of Interest

R.S. and A.H.‐C. are named as co‐inventors of the Systems Age framework which is the subject of a patent application. A.H.‐C. has received consulting fees from TruDiagnostic and FOXO Biosciences. R.S. has received consulting fees from TruDiagnostic, LongevityTech.fund and Cambrian BioPharma. The other authors declare no conflicts of interest.

## Supporting information


**Table S1:** Technical reliability of DNA methylation aging biomarkers across experimental conditions. Intraclass correlation coefficients (ICCs) for 18 DNA methylation–based aging biomarkers calculated across technical replicate datasets. Reliability is reported separately for slide location, Illumina 450 K arrays, Illumina EPIC (850 K) arrays, DNA extraction protocols, and pooled technical ICC estimates across all technical datasets.

**Table S2:** Biological reliability of DNA methylation aging biomarkers across short‐term physiological perturbations. Intraclass correlation coefficients (ICCs) for 18 DNA methylation–based aging biomarkers calculated from repeated biological samples collected under acute physiological and environmental conditions. Reliability estimates are reported separately for altitude exposure, meal consumption, acute stress, pollution exposure, and pooled biological ICCs across all biological datasets.

**Table S3:** Effect of immune cell composition adjustment on the biological reliability of DNA methylation aging biomarkers. Biological intraclass correlation coefficients (ICCs) following adjustment for estimated immune cell fractions. Results are presented for pooled analyses and stratified by individual perturbation conditions (meal, stress, pollution exposure, and altitude change) to evaluate the impact of immune cell composition adjustment on biological reliability.

**Table S4:** Relationship between epigenetic clock reliability and downstream analytic variability. Summary statistics linking epigenetic clock reliability with the stability of downstream analyses. Includes variability in prognostic associations derived from repeated resampling of technical replicates (ADNI dataset) and variability in estimated responsiveness to a vegan diet intervention across repeated biological measurements.

## Data Availability

All ICC values and clock scores are available through translage.io. Code to calculate all clocks except for OMICmAge and DNAmEMRAge is accessible at https://github.com/HigginsChenLab/methylCIPHER. Code to calculate OMICmAge, DNAmEMRAge, and underlying algorithms will be accessible via TruDiagnostic's DNAm Analysis Software. You can request access to the software at https://www.trudiagnostic.com/softwarerequest. We are currently working on a platform to download all TranslAGE harmonized data drawn from public sources.
